# Thrombotic thrombocytopenic purpura as a complication of pembrolizumab: a case report and literature Review

**DOI:** 10.1007/s00277-026-06907-3

**Published:** 2026-02-27

**Authors:** Gregory Palega, Ihab Ahmad Al-Rikabi, Malgorzata Kopaczek-Styczen, Cecilia Karlström

**Affiliations:** 1 Acute Care Unit, Department of Emergency Medicine and Internal Medicine, Karolinska University Hospital, Stockholm, Sweden; 2 Medical School, Karolinska Institutet, Stockholm, Sweden; 3 Department of Clinical Neuroscience, Karolinska Institutet, Stockholm, Sweden; 4 Department of Neurobiology, Care Sciences and Society, Karolinska Institutet, Stockholm, Sweden; 5 Department of Women’s and Children’s Health, Karolinska Institutet, Stockholm, Sweden; 6 Department of Hematology, Karolinska University Hospital, Stockholm, Sweden; 7 Department of Medicine Huddinge, Center for Hematology and Regenerative Medicine, Karolinska Institutet, Stockholm, Sweden

**Keywords:** Thrombotic thrombocytopenic purpura, TTP, Immune checkpoint inhibitors, ICI, Pembrolizumab, Case report

## Abstract

The success of immune checkpoint inhibitors (ICIs) has revolutionized oncology, with an increasing number of patients receiving treatment every year. However, this progress has been accompanied by a rise in immune-related adverse events (irAEs). One such irAE is thrombotic thrombocytopenic purpura (TTP), a rare and potentially life-threatening complication. This report presents a unique case of TTP following a single dose of pembrolizumab, a PD-1 inhibitor. A 76-year-old man with suspected advanced renal cell carcinoma received pembrolizumab as initial treatment. Eleven days later, the patient developed severe thrombocytopenia, bleeding problems, and hemolytic anemia. The following day, ADAMTS13 activity levels were measured at 3.8% (reference range: 40–130%), confirming the diagnosis of TTP. Given the patient’s poor overall condition and limited life expectancy, plasma exchange was not initiated. The patient passed away 15 days after receiving pembrolizumab. This case highlights that even a single dose of pembrolizumab can precipitate TTP, underscoring the need for clinician vigilance. A brief review of previously reported cases of PD-1 inhibitors associated with TTP is included.

## Introduction

The 2018 Nobel Prize in Physiology or Medicine was awarded to James P. Allison and Tasuku Honjo for discovering that inhibiting endogenous immune checkpoints can produce antitumor immunity [1, 2]. Immune checkpoint inhibitors (ICIs) are humanized monoclonal antibodies that block inhibitory pathways of the immune response, such as cytotoxic T-lymphocyte–associated protein 4 (CTLA-4) or programmed cell death protein-1 (PD-1) and its ligand, PD-L1. This blockade results in enhanced antitumor activity by unbridling the immune system [3]. Pembrolizumab, an anti-PD-1 IgG4 antibody, is widely used across multiple indications, including non-small cell lung cancer, advanced renal carcinoma, and melanoma [4]. Rarely pembrolizumab can cause immune-related adverse events (irAEs), which commonly affect the skin, gastrointestinal tract, and endocrine organs, and less often the nervous, hematopoietic, or urinary systems [5].

Among rare but life-threatening hematologic irAEs is acquired/immune thrombotic thrombocytopenic purpura (aTTP), a thrombotic microangiopathy caused by severe deficiency in ADAMTS13, a metalloprotease enzyme responsible for cleaving ultra-large von Willebrand factor (vWF) multimers [6, 7]. When ADAMTS13 activity decreases, these large vWF multimers accumulate and promote excessive platelet aggregation and formation of microthrombi in the microcirculation, with subsequent thrombocytopenia, hemolytic anemia, and organ damage [6, 7].

aTTP most commonly arises in the context of infection, autoimmune disease, or after exposure to certain medications, all of which can result in immune-mediated inhibition of ADAMTS13 [8]. aTTP can also occur due to certain cancers but must be differentiated from cancer-associated thrombotic microangiopathy (CA-TMA), which also causes thrombotic thrombocytopenia but lacks severe ADAMTS13 deficiency. Instead, CA-TMA is driven by tumor-related endothelial injury with resultant disseminated intravascular coagulation [9–12]. CA TMA’s clinical presentation is varied but can include thrombocytopenia, hemolytic anemia, schistocytes on blood smears, neurological symptoms (e.g., confusion, seizures), renal dysfunction, and fever [6, 7] making it similar to aTTP. However, an ADAMTS13 activity of < 10% and a normal coagulation profile support an aTTP diagnosis, whereas disseminated intravascular coagulation (DIC) features and poor response to plasma exchange supports a CA-TMA diagnosis [13]. Indeed, clinicians must correctly differentiate these two syndromes because the treatments differ substantially. In particular, early recognition of aTTP is critical because initiating treatment with plasma exchange, glucocorticoids and caplacizumab can reduce organ damage and has been proven to reduce mortality [14–16].

**Fig. 1:**
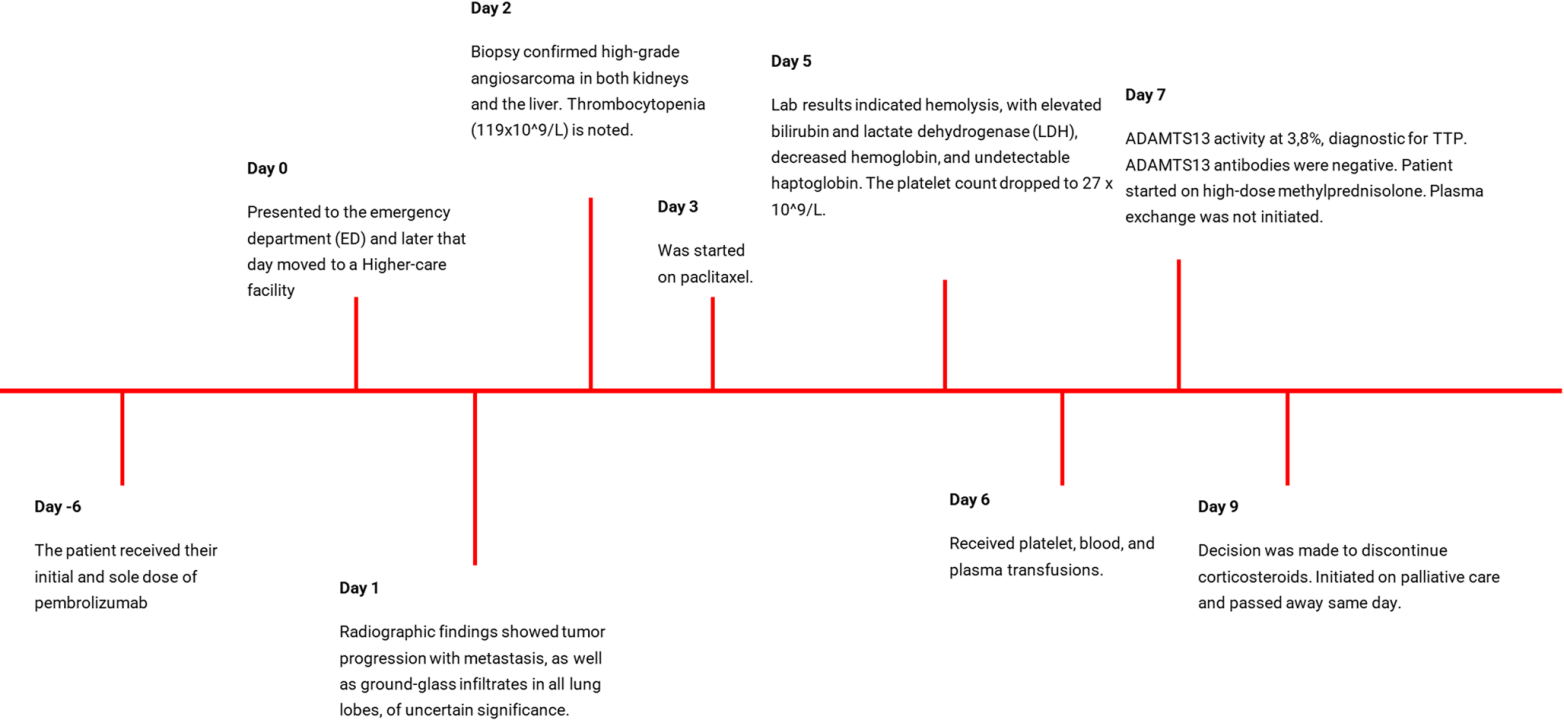
Timeline of the patient following a sole dose of pembrolizumab

The current report presents a unique case of aTTP occurring after a single dose of pembrolizumab (Fig. 1). While a few case reports have documented aTTP following the use of pembrolizumab, to our knowledge, this is one of few instances of aTTP developing after just one dose. In addition, a review of current case reports on PD-1 inhibitors associated with aTTP will also be provided to contextualize this case within the limited existing literature.

### Narrative

A 76-year-old man presented to his primary care physician with dyspnea, significant weight loss, and hemoptysis over the previous few weeks. His medical history was notable for hypertension and a 35-year smoking history. He also reported a family history of gastric cancer, although the specific type was unknown. Computer tomography (CT) revealed an advanced cystic renal tumor and possible metastases in the liver, abdomen, lungs, and submandibular glands. A biopsy was performed. However, due to extensive necrosis, a definitive histopathological diagnosis could not be established. Despite the nondiagnostic initial biopsy, a review of all the data at a multidisciplinary conference supported a high clinical suspicion of advanced renal cell carcinoma. This, plus the rapid clinical progression, prompted the initiation of treatment with pembrolizumab. Baseline laboratory values obtained prior to pembrolizumab administration showed low hemoglobin, but normal platelet counts and normal coagulation parameters. A repeat biopsy performed several days after pembrolizumab treatment revealed that the tumor was vascular in nature, which lowered the suspicion of advanced renal cell carcinoma (the PD-L1 status for this biopsy was not specified).

Approximately six days after receiving pembrolizumab, the patient presented to the emergency department (ED), of a university cancer center, with worsening dyspnea, hypoxia (oxygen saturation of 67% on room air), fever (38.8 °C), hematuria, and petechiae. On admission, his hemoglobin was 72 g/L, down from 80 just days before and despite transfusion of two units of packed red blood cells at that time. However, platelet counts and coagulation tests were within normal limits. The hematuria was suspected to have originated from the renal tumor. He was given two additional units of blood on admission.

On admission, the patient’s CRP and procalcitonin levels were 141 mg/L and 1.1 µg/L respectively. Chest CT revealed bilateral ground-glass infiltrates involving all lung lobes. These results, along with the rapid worsening of the patient’s clinical condition, prompted the initiation of an empirical treatment of intravenous piperacillin-tazobactam (Pip/Taz). In addition, 40 mg of prednisolone daily was administered to cover inflammatory pneumonitis.

Given his deteriorating condition, the patient was transferred to a higher level of care the same day. Blood cultures and β-glucan tests were negative. Radiographic findings revealed tumor progression with metastasis in the lungs, kidney, liver and mediastinal lymph nodes. A repeat biopsy on hospital day 2 was performed. Also on this day, the patient’s platelet levels began to drop, measured at 119 × 10^9/L, down from previously normal levels. On day 3, after the biopsy results revealed angiosarcoma, paclitaxel therapy (80 mg/m²) was administered due to the patient’s rapid deterioration.

On hospital days 4–5, laboratory findings indicated hemolysis, as supported by elevated bilirubin and lactate dehydrogenase (LDH), falling hemoglobin, and dropping haptoglobin levels (Table 1). By day 5, the platelet count had fallen further to 19 × 10^9/L, and the patient exhibited bleeding from multiple sites, including melena. Due to ongoing bleeding and thrombocytopenia, platelet transfusion was administered on day 5 but given his overall poor condition, gastroscopy was not performed. On day 6, tranexamic acid and an additional 2 units of platelets were administered. Subsequently, the consulting hematologist suggested acquired thrombotic thrombocytopenic purpura (aTTP) as a possible diagnosis, and ADAMTS13 activity testing was performed.

On hospital day 7, ADAMTS13 testing showed abnormally low activity at 3.8% (reference range 40–130%), confirming the diagnosis of aTTP. Peripheral blood smear examination demonstrated marked presence of schistocytes. At this point, ADAMTS13 antibody testing was negative. Given the patient’s overall poor condition, and in consultation with the attending oncologist, plasma exchange was not initiated. Instead, 120 mg of methylprednisolone was administered with the goal of improving the patient’s well-being. Unfortunately, after two days of steroid treatment with limited benefit, treatment was discontinued, and care was transitioned to palliation. The patient passed away later that day, on hospital day 9.

**Table 1:** Blood parameters of the patient before and during hospital stay. For days with multiple measurements, the lowest value is reported

Blood Parameters	Day − 11 (5 days before Pembro Adminsitration)	Day − 4 (2 days after Pembro Adminsitration)	Day 0 (ED Admission)	Day 1	Day 2	Day 3	Day 4	Day 5	Day 6	Day 7	Day 8	Day 9
Platelets (145–348) × 10^9/L	287	262	188	160	119	109	72	19	27	54	47	32
Nr of PLT Transfusion								1	1			
PK (INR) (< 1,3) sec	1,1	1,1		1,2		1,2	1,2	1,2	1,2	1,2		
APTT (20–30) sec	25	24		26			26	25	26	26		
Hemoglobin (134–170) g/L	88	80	72	80	87	73	72	72	75	76	82	78
Units of Blood transfusion given			2	1			2		2			
Creatinine (< 100) µmol/L			93	83	84	75	77	95	144	185	236	266
CRP (< 3) mg/L			146	185	163	103	95	110	133	129	127	91
WBC (3,5–8,8) x10^9/L			17,1	15,1	16,6	18,5	16,9	14,5	12	10,4	8,1	8,6
Lactate Dehydrogenase (< 4,3) mikrokat/L			9,5	9	10,9	11,1	15					
Bilirubin (< 26) mikromol/L			28	17	21	18	23	48	52	42	30	
Haptoglobin (0,24 − 1,9) g/L			0,96				0,29	< 0,1	< 0,1	< 0,1		
Fibrinogen (2,0–4,2) g/L							1,9	2,0	2,1	1,9		
ADAMTS13 Activity (40–130) %										3,8		
ADAMTS13 Antibodies (< 0,4) kBE/L										< 0,4		
Fecal Occult Blood (F-Hb)								Positive				

*Pembro=Pembrolizumab*

### Patient perspective

The patient was diagnosed with highly malignant angiosarcoma two days after presenting to the emergency department. Despite the severity of the diagnosis, the patient initially expressed a strong desire to pursue treatment and was fully aware of the precarious nature of the situation. However, after seven additional days, the patient was actively involved in the decision to discontinue further therapy.

## Discussion

This patient was diagnosed with thrombotic thrombocytopenic purpura (TTP) 13 days after a single dose of pembrolizumab. The diagnosis of aTTP was confirmed by low ADAMTS13 activity. Given the rapid onset of TTP symptoms eleven days after pembrolizumab administration, it is most likely that the immune checkpoint inhibitor played a central role in triggering aTTP, which is consistent with known hematologic toxicities associated with ICIs [5, 17–21]. Although this patient tested negative for anti-ADAMTS13 antibodies, it is not uncommon for aTTP patients to present with negative antibodies, at least initially.

There are other possible causes for aTTP, or reduced ADAMTS13 activity, which should be considered. First, certain medications can trigger aTTP, but we thought the particular medications in this case to be less likely causative than Pembrolizumab. For example, paclitaxel, which was first administered after the onset of clinical bleeding and thrombocytopenia, was less likely to be a culprit in this case due to the timing and because paclitaxel is rarely associated with thrombocytopenia-related complications such as aTTP [22]. Piperacillin was also considered an unlikely cause of aTTP in this case because of its exceedingly rare association, reported only once by Yata et al. (2000) in a case in which the patient also exhibited hemorrhagic colitis and a skin eruption. These latter features were absent in the present case [23]. No other medications in this case had plausible timing or clinically likely associations with aTTP.

Second, although congenital TTP (cTTP) can present later in life [24], this patient’s advanced age, the timing of recent pembrolizumab use, and the known immune-related adverse effects of ICIs [17, 18, 21, 25], make cTTP less likely.

Third, decreased activity of ADAMTS13 can sometimes be seen with decreased hepatic function. However, this seems less likely in our case since the normal liver function tests as assessed by PK(INR) and fibrinogen suggest adequate hepatic function.

Fourth, regarding CA-TMA and its ability to mimic many signs and symptoms of aTTP. As mentioned in the introduction, CA‑TMA typically does not show severe ADAMTS13 deficiency (< 10%) and is instead often accompanied by DIC or marrow infiltration (leukoerythroblastosis). In this case, the persistently normal PT and PTT levels throughout the hospital course make DIC very unlikely, and no blast cells were seen in circulation [13, 26].

Fifth, regarding the cancer itself causing aTTP, angiosarcoma has not been reported to cause aTTP. It can precipitate MAHA/TMA via a Kasabach-Merritt-type consumptive coagulopathy, but the normal PT and PTT plus the severe ADAMTS13 deficiency in this case is much more consistent with aTTP [26, 27]. In particular, the documented severe ADAMTS13 deficiency together with the short latency (11 days) after PD‑1 blockade, a recognized trigger of hematologic irAEs, provides a pathophysiologic fit for ICI‑triggered aTTP.

### Treatments

The standard treatment for aTTP includes plasma exchange, rituximab, corticosteroids, and caplacizumab [6, 14]. In this case, despite plasma exchange being recommended by the hematologist, the attending oncologist decided not to initiate plasma exchange due to the patient’s instability and general poor condition. There was no mention of consideration of treatment with caplacizumab and rituximab in this patient’s record. Instead, high-dose methylprednisolone was attempted, but provided limited benefit.

Platelet transfusions, while typically used in cases of severe thrombocytopenia, can worsen TTP by accelerating platelet aggregation [28]. In this case, platelet transfusion was given due to ongoing bleeding before the ADAMTS13 test had returned and in the context of already severe thrombocytopenia. In this case, we feel it was unlikely that there was a clinical deterioration because of the platelet transfusion.

### Autoantibody negativity

In our case, the patient tested negative for anti-ADAMTS13 antibodies. These antibodies would be expected to be present as part of the pathophysiology of aTTP. However, a literature review shows that aTTP can initially present without detectable ADAMTS13 antibodies [29].

A possible explanation for the negative anti-ADAMTS13 antibody result could be a technical issue with the assay used to detect the antibodies. Another explanation could be low antibody levels early in the course of the disease. In fact, studies have documented that antibody negativity can occur, particularly in the early stages of immune-related TTP (iTTP) [30, 31]. A retrospective study of all ADAMTS13 activity tests performed in a single center [30] revealed that, among patients with low ADAMTS13 activity and no detectable antibodies, the majority later tested positive for anti-ADAMTS13 antibodies in subsequent assays. However, not all patients demonstrated this progression. The authors suggested that most initial negative antibody tests resulted from low or transient autoantibody production at the time of initial testing.

Simon et al. [31] conducted a similar retrospective study of patients with aTTP who had ADAMTS13 deficiency at presentation. They reported that 21% of those who initially tested negative for anti-ADAMTS13 antibodies later tested positive during follow-up. With either of these studies, it could not be ascertained whether initial negative antibody tests in those who later tested positive resulted from a test sensitivity problem or de facto negative (or extremely low) serum antibody levels.

Future case reports examining ICI-induced aTTP would benefit greatly from routine testing for anti-ADAMTS13 antibodies at multiple points in time to explore the relationship between autoimmunity and the development of thrombocytopenia in this context. Such testing could enhance our understanding of the disease mechanism and provide important prognostic insights, especially as the role of autoimmune activation in ICI-induced aTTP continues to be investigated.

## Literature review

As of October 2025, we conducted a literature review on acquired thrombotic thrombocytopenic purpura (aTTP) secondary to immunotherapy targeting PD1. We utilized PubMed and Web of Science and identified 14 case reports on the subject, including ours. These reports, summarized in Table 2, provide key insights into the clinical manifestations, diagnosis, treatment, and outcomes of TTP in patients treated with PD-1 inhibitors. We searched for all monoclonal antibodies targeting PD1 but found reports only in patients receiving pembrolizumab or nivolumab, often in combination with other agents. Notably, five cases involved concurrent treatment with ipilimumab and nivolumab, complicating attribution of causality, as ipilimumab-induced aTTP has also been previously documented [32, 33]. In a previous review of case reports of pembrolizumab-induced aTTP by Gilbar et al. [34], only three case reports had been published, indicating that the body of evidence on this adverse event is still expanding.

**Table 2:** Overview of reported cases of thrombotic thrombocytopenic purpura associated with PD-1 inhibitors

Author & Year	Age & Sex	Type of Cancer	Cancer Treatment	Timing of TTP from last regimen	Clinical Presentation of TTP	ADAMTS13 Activity	ADAMTS13 antibodies	Treatment of TTP	Outcome
Dickey et al. 2020 [35]	60-year F	NSCLC	Pembrolizumab × 5 cycles - every 3 weeks; Total dose 1000 mg	14 days	Chest pain, pyrexia & Dyspnea	3%	Not reported	PE x 5, methylprednisolone, prednisolone	Initially, complete remission. 4 days later probable relapse. Abstained from further treatment. Death.
De Filippis et al. 2021 [36]	61-year M	NSCLC	Pembrolizumab, carboplatin, paclitaxel × 5 cycles then pembrolizumab × 2 cycles; Total dose 1400 mg	46 days	Jaundice, scleral icterus, costovertebral tenderness (probably due to underlying tumor)	10–12%	Positive	PE x 5, methylprednisolone, prednisolone	After stabilization, patient declined further treatment and died out-of-hospital.
Nelson et al. 2022 [37]	68-year M	Metastatic urothelial carcinoma	Pembrolizumab x 3	Not Reported	Confusion, SOB, Mild Tachycardia	8%	Positive	Not initiated	Death
Sharma et al. 2023 [38]	69-year F	Stage IV lung cancer	Pembrolizumab x 2	Not Reported	SOB, 3 days later hypoxia, hypotension, limb paresis & facial drop	0.56 IU/mL (0.68–1.63 IU/mL)	Not reported	Not initiated	Death
Kozak et al. 2023 [39]	56-year M	Metastatic Lung Adenocarcinoma	Pemetrexed, carboplatin, pembrolizumab x 4 cycles. 1 month later 5th maintenance dose of pemetrexed and pembrolizumab.	2 months	Episode 1: Altered Mental Status & Thrombocytopenia. Episode 2: Fatigue, Chills & Severe Diarrhea (positive C. difficile toxin B).	Episode 1: not measured. Episode 2:<5%	Positive	Episode 1: PE x 6, corticosteroids Episode 2: PE x 7, high dose dexamethasone, Rituximab x 4	Complete remission. No reported relapse of TTP.
Luong et al. 2024 [40]	73-year F	Metastatic pancreatic adenocarcinoma	Pembrolizumab x 1	1 month	DKA & Diarrhea. Thrombocytopenia & hemolytic anemia.	No lab done before PE. After PE: 48%	Not reported	PE x 1, corticosteroids	Moved to home hospice. Unclear outcome
This case	76-year M	Angiosarcoma (suspected advanced renal cell carcinoma)	Pembrolizumab x 1	11 days	Dyspnea, Fever, hematuria, Petechia	3%	Negative	Methylprednisolone, prednisolone	Death
Parekh et al. 2025 [41]	Mid-40s M	Metastatic melanoma stage IV	Ipilimumab & nivolumab x 3	2 weeks	Altered mental status, AKI, Fever, Right gaze deviation,	< 5%	Not reported	PE x 3, Methylprednisolone, Caplacizumab, Rituximab.	Complete remission. No reported relapse of TTP.
Yoshida et al. 2024 [42]	78-year M	Reconstructed gastric tube adenocarcinoma	SOX x 3, discontinued. Nivolumab x 15.	2 weeks	No symptoms. Blood test indicated thrombocytopenia and hemolytic anemia.	1%	Not reported.	PE x 2, methylprednisolone, prednisolone, Rituximab	Complete remission. No reported relapse of TTP.
Mullally et al. 2022 [43]	Early 50s M	Metastatic melanoma	Ipilimumab & nivolumab x 1	10 days	AKI, subacute infarction, seizure, petechia.	< 5%	Not reported	PE x 1, methylprednisolone.	Death
Ali et al. 2020 [44]	46-year M	Metastatic stage IV clear cell renal cell carcinoma	Ipilimumab & nivolumab x 4	2 weeks	Intermittent fever, back pain, hematuria, AKI	< 2%	Not reported	PE x 11, prednisone, rituximab, caplacizumab	Complete remission. No reported relapse of TTP.
Lancelot et al. 2020 [45]	56-year F	Stage IV malignant melanoma	Ipilimumab & nivolumab x 4	8 weeks	Dizziness, weakness	< 5%	Not reported	Episode 1: IVIG (for suspected ITP), PE x 21, rituximab, prednisone. Episode 2: dexamethasone, bortezomib, PE x 17, Outpatient: PE x 3, prednisone	Death
Gergi et al. 2020 [46]	51-year F	Stage IIIc anal carcinoma	5-fluorouracil and mitomycin. Adjuvant nivolumab x 3	41 days	Persistent nausea, vomiting, diarrhea, worsening dyspnea on exertion, hematuria, and headache	9%	Not reported	PE x 1 month, prednisone, rituximab,	Complete remission. No reported relapse of TTP.
Yousesf et al. 2018 [47]	42-year F	Metastatic renal cell carcinoma	Ipilimumab (1 mg/kg) & nivolumab (3 mg/kg) x 1	9 days	Altered mental status, slurred speech, and fever	3%	Not reported	PE x 8, methylprednisolone, prednisone, rituximab	Complete remission. No reported relapse of TTP.

*AKI= Acute Kidney Injury. SOB= Shortness of Breath. NSCLC= Non-Small Cell Lung Cancer. DKA= Diabetes Ketoacidosis. PE= Plasma Exchange*

The median age of patients across the reviewed cases was approximately 60 years, with a range of circa 35 years. The median time from the last dose of immunotherapy to the onset of TTP symptoms was circa 27 days, although the earliest occurred 9 days and the latest occurred 8 weeks later. Among the patients reported, eight were male, and six were female. In terms of treatment cycles, the number of cycles administered ranged from 1 to 15 cycles, with Youssef et al. noting the highest number of cycles. However, details on the dosages were often not included in the case reports, making it difficult to draw firm conclusions on the role of dose in triggering hematologic complications. Gilbar et al. [34] speculated that the shift from bodyweight-based dosing to flat dosing for pembrolizumab administration could explain an increase in adverse effects, as the flat dose might be disproportionately high for many patients, potentially contributing to an increased risk of hematologic toxicity.

We identified seven articles describing nivolumab-associated aTTP and seven describing pembrolizumab-associated aTTP. Among the nivolumab-related cases, two resulted in death, whereas five deaths were reported among patients with pembrolizumab-associated aTTP. This notable difference might be due to chance or partially explained by age differences between the groups, with a mean age of approximately 66 years among pembrolizumab-treated patients compared to about 52 years in the nivolumab group. Another plausible factor is the variation in the use of plasma exchange, as all patients receiving nivolumab underwent multiple sessions, whereas only four patients treated with pembrolizumab received this intervention. Nonetheless, it remains unlikely that there are intrinsic differences in irAEs between pembrolizumab and nivolumab [48, 49].

The clinical presentations of aTTP varied across the reported cases. This is in line with the conclusions drawn from a review of case reports on ICI-induced TTP by Yikilmaz et al. [32]. One interesting case by Luong et al. [40] involved the development of DKA due to a new onset of type 1 diabetes mellitus (DM1) alongside aTTP after pembrolizumab treatment, indicating an autoimmune mechanism. Pembrolizumab-induced autoimmune diabetes has been described in the literature and has been suggested to be caused by a dysregulation of cytotoxic T-lymphocytes [50, 51]. However, whether autoantibodies were responsible for the observed diabetes and aTTP in the case by Luong et al. [40] remains undetermined [52], highlighting the need for further research into the immune mechanisms at play in ICI-induced adverse effects.

Another unique case was reported by Lancelot et al. [45], describing a patient treated with ipilimumab and nivolumab who experienced three episodes of plasma exchange due to recurrent aTTP. The patient also received rituximab and bortezomib, which together were expected to reduce autoreactive B lymphocytes and plasma cells, thereby decreasing antibodies targeting ADAMTS13. The authors speculated that the lack of response to these therapies may indicate that TTP occurring in patients receiving immune checkpoint inhibitors differs mechanistically from other forms of aTTP.

ADAMTS13 activity, as measured through enzyme assays, was low in almost all cases, confirming the diagnosis of aTTP. However, many authors did not measure or report anti-ADAMTS13 antibodies. In three case reports, the authors tested for anti-ADAMTS13 antibodies and found them positive, while the others did not report antibody testing. Similar findings were observed in other case reports on ICI-induced aTTP [32].

Treatment strategies varied, with plasma exchange and corticosteroids being the most commonly administered therapies, as per the International Society on Thrombosis and Haemostasis (ISTH) clinical guidelines for aTTP [14, 16]. Recently, caplacizumab has emerged as a promising new therapy for aTTP and is recommended to be used alongside PE in acute cases. However, ISTH has emphasized that, although caplacizumab may offer significant therapeutic benefits, its availability remains limited, and many clinicians are unfamiliar with its use and the necessary monitoring protocols [16]. As evident by our review and the review by Yikilmaz et al. [32], few cases in the literature initiated caplacizumab therapy, highlighting that its integration into routine practice is still developing.

Eleven cases initiated PE, whereas three cases did not, with the decision to forgo PE being made in consultation with the patient, particularly in cases where the patients were in poor clinical condition. For example, Nelson et al. [37] reported a patient who declined all treatments, including PE and corticosteroids, due to prior comorbidities and a decision to focus on comfort care. Similarly, Sharma et al. [38] described a patient who chose not to pursue further treatment after the diagnosis of TTP. In our case, the decision was made not to initiate PE, also due to poor overall condition, and instead to try high-dose methylprednisolone. However, this was discontinued, with the patient’s consent, after three days due to the patient’s rapidly declining condition.

The ISTH strongly recommends PE in combination with corticosteroids for patients experiencing a first acute episode of aTTP, as well as those experiencing a relapse [16]. Evidence shows a 90% survival rate following PE after the first episode, with treatment to be administered daily until signs of organ damage resolve and platelet counts stabilize. This survival rate underscores the importance of early diagnosis and timely initiation of PE [6].

Among the 14 patients reviewed, seven patients died, which reflects the high mortality associated with aTTP secondary to PD-1 inhibitors. Six patients achieved complete remission, while the outcome for one patient remains unclear. It is important to note that all patients had multiple comorbidities, making it difficult to isolate the contribution of aTTP alone in the patients’ poor outcomes. In several cases, the presence of cancer, severe infections, and other preexisting health conditions likely contributed to a poor outcome. This review suggests that early recognition and treatment of aTTP may be important for improving patient prognosis, although the limited data available do not allow for definitive conclusions.

## Conclusion

In conclusion, this case highlights the importance of recognizing pembrolizumab-induced aTTP as a cause of thrombocytopenia, even after a single dose. This review of the current case reports highlights the need to be vigilant for ICI-induced TTP and to consider the potential autoimmune pathophysiology as soon as the diagnosis is established. Early testing for anti-ADAMTS13 antibodies can benefit both researchers’ and clinicians’ knowledge base as well as inform individual patient treatment decisions. Since treatment for aTTP is distinct from other causes of thrombocytopenia, and effective treatments can be offered, physicians must recognize aTTP early to optimize the chances for treatment success.

## Data Availability

No datasets were generated or analysed during the current study.
